# Characteristics Associated with Infant Feeding with Both Breast Milk and Formula Milk

**DOI:** 10.3390/nu18111726

**Published:** 2026-05-28

**Authors:** Kenta Watakabe, Sayaka Kawada, Shin Horiuchi, Rin Asahiro, Airi Tanaka, Kyoka Tei, Yayoi Murano, Tomoyuki Nakazawa, Ken Sakamaki, Hiromichi Shoji, Daisuke Yoneoka

**Affiliations:** 1 Division of Pediatrics, Tokyo Metropolitan Toshima Hospital, 33-1 Sakaecho Itabashiku, Tokyo 173-0015, Japan; 2 Department of Pediatrics, Juntendo University, 3-1-1 Hongo Bunkyoku, Tokyo 113-0033, Japan; 3 Division of Obstetrics and Gynecology, Tokyo Metropolitan Toshima Hospital, 33-1 Sakaecho Itabashiku, Tokyo 173-0015, Japan; 4 Graduate School of Medicine, University of Tokyo, 7-3-1 Hongo Bunkyoku, Tokyo 113-0033, Japan; blue.sky.sea.dy@gmail.com

**Keywords:** breastfeeding, breast feeding, human milk, formula milk, lactation

## Abstract

**Background**: Breastfeeding benefits mothers and infants, and the promotion of breastfeeding is important. Feeding strategies include exclusive breastfeeding, feeding with both human milk and formula, and exclusive formula feeding. **Objectives**: This study was conducted to clarify the actual situation by assuming that mixed feeding comprises several groups with different characteristics. At the same time, the study also aimed to clarify the factors associated with breastfeeding. **Methods**: Single-term infants without underlying disease born at Tokyo Metropolitan Hospital between 2019 and 2024 participated in this study. The distribution of formula intake among infants receiving both human milk and formula was analyzed using a Gaussian mixture model, and the optimal number of distribution components was calculated using the Bayesian information criterion. Using linear regression analysis, factors associated with formula intake were identified. **Results**: A total of 2628 participants (exclusive breastfeeding, 842 (32.0%); mixed feeding with human milk and formula, 1496 (56.9%); and exclusive formula feeding, 290 (11.0%)) were included in the study. Linear regression analysis showed that the factors associated with amount of formula intake were late preterm birth (coefficient 39.7, *p* < 0.01), maternal age (reference under 30 y, age ≥ 30 y and <35 y coefficient 6.3, *p* = 0.66, age ≥ 35 y and <40 y coefficient 45.5, *p* < 0.01, age ≥ 40 y coefficient 106.9, *p* < 0.01) and delivery mode (cesarean section, coefficient 53.6, *p* < 0.01). **Conclusions**: Feeding strategies involving both human and formula milk are not homogeneous, and interventions should be developed based on these differences. Moreover, several factors were found to be associated with breastfeeding, which may help promote breastfeeding.

## 1. Introduction

Breastfeeding benefits both mothers and infants, and the World Health Organization (WHO) recommends exclusive breastfeeding until six months of age [1]. Substantial evidence demonstrates that breastfeeding not only reduces the risk of infectious diseases in infancy but also contributes to long-term health outcomes, such as improved cognitive development and a lower likelihood of obesity and diabetes in childhood. Moreover, recent evidence from research suggests that reproductive history and long-term maternal health outcomes, including obesity and hypertension, are closely linked to breastfeeding practices, highlighting the importance of understanding maternal and infant feeding behaviors in a broader life-course perspective [2]. For mothers, breastfeeding has been associated with a reduced incidence of several non-communicable diseases, including breast cancer [3]. These studies support a life-course perspective on infant feeding by showing that reproductive history is linked to women’s long-term health. These findings suggest that infant feeding practices should be understood not only in relation to immediate neonatal outcomes but also in the broader context of maternal health trajectories across the life course. This perspective further strengthens the rationale for examining heterogeneity within mixed feeding groups and for understanding maternal and infant feeding behaviors in a more comprehensive framework.

Scaling up breastfeeding prevented 823,000 child deaths and 20,000 annual deaths from breast cancer [3]. Therefore, the promotion of breastfeeding is an important issue worldwide and a major challenge for healthcare providers. WHO promotes breastfeeding through several actions, including World Breastfeeding Week [4]; providing appropriate support to those who really need it is an important issue.

Despite these well-established benefits, breastfeeding rates remain suboptimal in many countries. Barriers to breastfeeding are investigated in several studies, and factors including maternal smoking and cesarean section have been identified [1]. The following overview studied barriers more comprehensively and defined barriers into four categories: therapeutic and care interventions; support networks and education; maternal-infant health issues; and societal and environmental context [3]. The authors highlight the importance of tailored support. Therefore, in this study, instead of identifying a single factor, we aimed to address mothers requiring additional support for breastfeeding more comprehensively.

In Japan, the Ministry of Health, Labor and Welfare distributes a manual for breastfeeding, which encourages educational classes for breastfeeding and helps to provide breastfeeding support to anyone who requires it [4]. However, exclusive breastfeeding accounted for 49.7%, 45.2%, and 52.6% at 0, 1, and 2 months after birth, respectively, while infants fed with a mixture of breastfeeding and formula milk represented 48.4%, 51.3%, and 40.9% [5]. There are 61 “Baby Friendly Hospitals” which successfully promote breastfeeding (approximately 4% of births in Japan occur in these hospitals), which is a relatively low number compared to other developed countries [6]. However, despite the fact that the timing of education for breastfeeding and interventions for successful breastfeeding varies by institution, the breastfeeding situation is improving [4].

Japan is a developed country, and the Mother and Child Health Handbook, which includes all perinatal information, is distributed to all mothers who undergo maternal health checkups and/or deliveries in Japan. In the handbook, health care providers record the feeding status as one of the following: exclusive breastfeeding, a mixture of breastfeeding and formula feeding, or exclusive formula feeding. The WHO classification of breastfeeding is similar, but is defined as exclusive breastfeeding, any breastfeeding, and formula feeding [7]. However, we questioned whether the feeding status classified as a mixture of breastfeeding and formula feeding, or any breastfeeding, represented a single group with shared characteristics. In this study, we assumed that these groups contained multiple subgroups with distinct characteristics and aimed to identify them. Understanding these characteristics may contribute to the development of effective breastfeeding support.

## 2. Materials and Methods

This retrospective study extracted the following information from the medical records: maternal age, delivery mode (normal delivery vs. cesarean section), gestational age, birth weight, sex, details of feeding at the one-month checkup (exclusive breastfeeding, breastfeeding and formula feeding, formula feeding only, and amount of formula), presence of congenital disease, and hospital stay at the one-month checkup. The study period was from January 2019 to January 2024. Participant characteristics were compared using the chi-square test for categorical variables and analysis of variance (ANOVA) for continuous variables.

First, based on our hypothesis that mothers who feed with both human milk and formula milk have several characteristics, participants who used the same feeding strategy were identified. The amounts of breast milk and formula milk per weight (kg) were calculated, and histograms were plotted. The number of components (i.e., the number of peaks) in the distribution was estimated using a Gaussian mixture model (GMM), a probabilistic clustering method that assumes the observed distribution is composed of several Gaussian components, each representing a latent subgroup. Gaussian mixture models offer the advantage of soft clustering, allowing each data point to belong to multiple clusters with varying probabilities rather than being rigidly assigned to a single group, while also flexibly capturing clusters of different shapes. The number of components ranged from one to five, and the Bayesian information criterion (BIC) was used to select the optimal number of components. Lower BIC values were interpreted as indicating a better model.

Next, to understand the characteristics of feeding with less formula milk and therefore more human milk, which reflects greater reliance on human milk, a linear regression analysis was performed. The dependent variable was the amount of formula milk, while independent variables included maternal age (<30 y, ≥30 y and <35 y, ≥35 y and <40 y, ≥40 y), delivery mode (normal delivery, cesarean section), and early term delivery (37 and 38, or >39 weeks of gestational age) [8], low birth weight (<2500 g), and infant sex. Because the relationship between these variables and feeding volume may be complex, the backward stepwise method was used to detect significant variables, avoiding overfitting. The same analysis was also performed with the amount of human milk. In these analyses, infants fed with human milk only and those with both formula milk and human milk were included.

Statistical analyses were performed using R software, version 4.4.2. Statistical significance was defined as *p* < 0.05. This study was approved by the Ethics Committee of Tokyo Metropolitan Toshima Hospital (approval no. Rin-Jin 6-85; approval date 10 February 2025). Data were extracted anonymously, and patient informed consent was not required. The study was conducted in accordance with the Declaration of Helsinki, and the human rights of the subjects were considered.

## 3. Results

### 3.1. Participants

#### 3.1.1. Number of Participants

**Figure 1:**
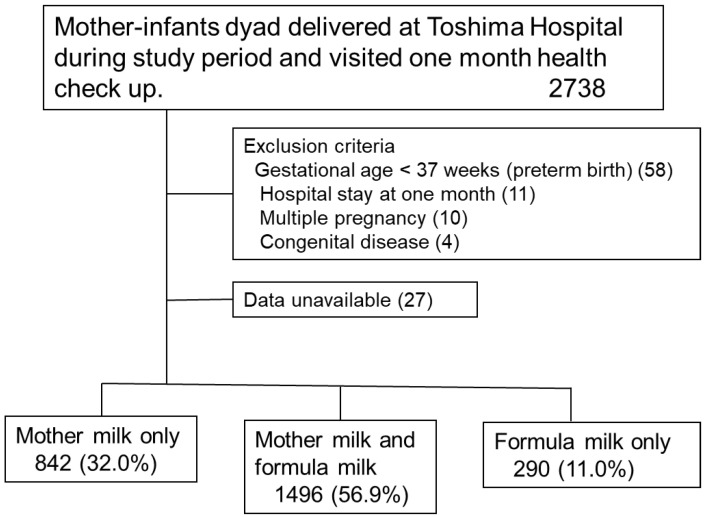
Study participants.

A total of 2738 deliveries occurred during the study period. Infants with preterm birth, a hospital stay of one month or less, or congenital diseases, and multiple pregnancies were excluded from the study to ensure a homogeneous study population. Of the remaining 2655 eligible mother–infant dyads, 27 (1.0%) were excluded because of insufficient or missing data. The final group of 2628 mother–infant dyads comprised 842 infants fed only human milk, 1496 fed both human milk and formula milk, and 290 fed formula milk only (Figure 1).

#### 3.1.2. Characteristics

The participating infants included 1337 (50.9%) boys, and the mean gestational age at birth was 39.3 ± 1.1 weeks. A total of 2105 (80.1%) infants were delivered via normal delivery, whereas 523 (19.9%) infants were delivered via cesarean section. The mean maternal age was 32.2 ± 5.2 years. Mean birth weight, body length, and head circumference at birth were 3068.1 ± 368.2 g, 48.7 ± 1.8 cm, and 33.5 ± 1.3 cm, respectively.

Several maternal and infant characteristics differed significantly among the three feeding groups (Table 1).

### 3.2. Details of Feeding Strategy

#### 3.2.1. Distribution of Formula Milk Feeding

**Figure 2:**
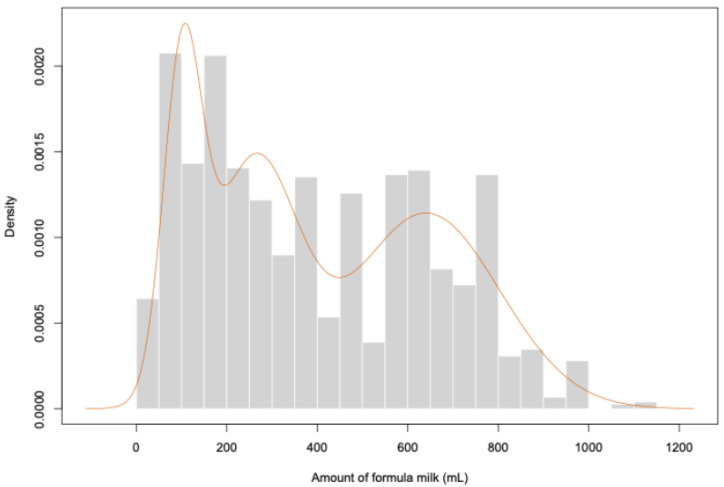
Gaussian mixture model for formula milk volume. Grey bar and orange line indicate the histogram of empirical probability and the GMM-estimated density plot, respectively.

For infants fed with both human milk and formula milk, Figure 2 shows the estimated density curve. The GMM showed three peaks based on the BIC, indicating that the fed milk volume can be decomposed into three underlying clusters. The GMM identified three distinct peaks, suggesting the presence of three latent subgroups within the mixed-feeding population.

#### 3.2.2. Regression Analysis

The factors associated with the amount of formula milk for infants fed with human milk only, and both human milk and formula milk, were analyzed using linear regression (Table 2). Early-term delivery, advanced maternal age, and cesarean section were associated with increased formula milk consumption. There was no association with low birth weight or infant sex.

The same analysis was performed with the amount of formula milk per weight at the one-month health check-up, and variables associated with the outcome were advanced maternal age and cesarean section. There was no association with early-term delivery, low birth weight, or infant sex (Table 3).

## 4. Discussion

The present study showed that the group using a feeding strategy of both breast milk and formula milk is not a single homogeneous population. Instead, this group comprises multiple subgroups with distinct characteristics, which, to the best of our knowledge, have not been reported before. Regression analysis detected factors associated with successful breastfeeding practices. While these factors are consistent with those previously identified, this study specifically found them to be associated with the amount of formula consumed.

The benefits of breastfeeding are well known, and promoting breastfeeding is an important issue worldwide. The present study introduces the concept of populations with different characteristics among infants fed with both human milk and formula milk: one group is fed with a small amount of formula milk, and the other group is fed with a large amount of formula milk. These patterns likely reflect different underlying motivations and circumstances.

Infants fed a small amount of formula milk are suggested to belong to at least two subgroups. In one subgroup, infants may be fed by guardians other than their mothers, and in the other, formula milk may be intentionally used to help infants sleep longer and to allow mothers more time to rest, because formula milk has a longer gastric emptying time [9,10]. Understanding these groups is important because, according to the Ministry of Health, Labour and Welfare, the number of Japanese women who work is increasing, and more fathers are taking childcare leave. The number of working women reached 27,930,000 in 2023, accounting for 45.1% of the working population [11], and the Cabinet Office announced a recent sharp increase in the number of fathers taking childcare leave [12]. Therefore, these populations are likely to continue to grow and have become increasingly important in recent years. Mothers of infants being fed a small amount of formula milk did not require breastfeeding support because their milk production was maintained. Moreover, studies have shown that sufficient rest and relaxation are important for breastfeeding outcomes [13,14], and that there are benefits from fathers’ involvement in childcare [15]. However, the reasons underlying this feeding practice remain unclear, and further qualitative research is needed. Furthermore, this population requires additional investigation, because, although the WHO recommends exclusive breastfeeding until six months of age, one study suggests that limited early cow milk exposure may prevent milk allergy in the future [16]. Considering these potential benefits, as well as the possible benefits for mothers and those from father–infant attachment mentioned above, this population needs to be studied further.

By contrast, mothers of infants fed large amounts of formula milk seem to produce insufficient amounts of milk. The major reason for discontinuing breastfeeding is the perception of an insufficient milk supply, which occurs because of poor breastfeeding initiation and/or insufficient breastfeeding education [17]. Breastfeeding failure is associated with low self-efficacy and poor infant sucking ability [16]. In the present study, early-term delivery, maternal age, and delivery mode were identified as factors associated with poor breastfeeding. A previous study reported that factors associated with breastfeeding included primiparity and high educational level, whereas those associated with formula feeding included single marital status, multiparity, and cesarean section [18]. Healthcare providers should understand these factors and provide sufficient support to those who need it. Support from healthcare professionals is effective for breastfeeding [8], and effective support strategies include skin-to-skin contact [19], support from health care providers [20], and early initiation of breastfeeding [21]. However, the quality of formula milk is improving, and since a baby’s healthy development is of the utmost importance, we must not overlook the necessity of supplementing with formula [22,23].

Few studies report on feeding practices involving both human milk and formula milk, although it has been established as one of the feeding strategies in infancy [24], and reasons for choosing the strategy are assessed as perceived necessity, perceived choice, and perceived pressure. [25]. Within these categories, feeding smaller amounts of formula milk is perceived as a choice, whereas feeding larger amounts is perceived as a necessity. However, in the future, assessment based on our category may be helpful for effective breastfeeding promotion.

There are several limitations in this study. Firstly, we could not assess other factors influencing breastfeeding, including socioeconomic status, race, and community situation [26,27]. Secondly, the study period includes the coronavirus disease 2019 (COVID-19) pandemic, which may have affected changes in the breastfeeding situation. The COVID-19 pandemic altered information sources on health-seeking [28], and further evaluation is needed to understand this issue better. Thirdly, this study is a retrospective single-center study. For future studies, prospective and multicenter studies are needed. Moreover, factors including educational programs, institutional policies, and other factors that affect breastfeeding are required. Lastly, we relied on backward stepwise selection for variable selection. Although this approach was appropriate for the exploratory nature of our analysis, it is known to potentially introduce model instability. Application of alternative variable selection methods, such as LASSO, elastic net, or model averaging, to confirm the robustness of the identified predictors represents an important direction for future work.

To the best of our knowledge, the present study is the first to characterize subgroups with different characteristics among mothers who fed both human milk and formula milk. Our findings highlight that while some mothers intentionally incorporate formula milk into their feeding strategies, others do so because of breastfeeding failure and may benefit from additional support. Recognizing these distinctions is essential for healthcare professionals aiming to provide individualized and efficient breastfeeding support. By understanding the diverse backgrounds and needs of mixed-feeding mothers, healthcare providers can better tailor interventions and contribute to improved breastfeeding outcomes at the population level. For further studies, these subgroups should be examined in depth with qualitative assessments and longitudinal follow-up, which investigates changes in future feeding patterns, as required.

## Figures and Tables

**Table 1:** Feeding group characteristics.

	Human Milk Only	Human Milk and Formula Milk	Formula Milk Only	*p*-Value ^1^
Maternal age (y)	31.8 ± 5.1	32.6 ± 5.2	31.7 ± 5.9	<0.01
Delivery method (normal delivery (%))	698 (82.9)	1193 (79.8)	214 (80.1)	<0.01
Gestational age (weeks)	38.9 ± 1.1	39.0 ± 1.1	38.8 ± 1.2	<0.05
Birth weight at birth (g)	3053.3 ± 360.1	3083.1 ± 366.1	3039.4 ± 398.7	0.06
Body length at birth (cm)	48.6 ± 1.8	48.8 ± 1.8	48.4 ± 1.9	<0.05
Head circumference at birth (cm)	33.4 ± 1.3	33.5 ± 1.3	33.4 ± 1.3	<0.05

^1^ Continuous variables, ANOVA; categorical variables, chi-square test.

**Table 2:** Factors associated with volume of formula milk (mL).

		Coefficient	95% CI	*p*-Value
Early term delivery		39.7	15.3–64.1	<0.01
Maternal age	<30 y			
	≥30 y and <35 y	6.3	−21.5–34.2	0.66
	≥35 y and <40 y	45.5	15.0–76.1	<0.01
	≥40 y	106.9	56.5–157.3	<0.01
Delivery method (cesarian section)		53.6	23.5–83.6	<0.01

CI: confidence interval.

**Table 3:** Factors associated with volume of formula milk (mL/kg).

		Coefficient	95% CI	*p*-Value
Maternal age	<30 y			
	≥30 y and <35 y	6.3	−7.6–11.0	0.72
	≥35 y and <40 y	13.4	3.26–23.6	<0.05
	≥40 y	35.8	19.0–52.6	<0.01
Delivery method (cesarian section)		13.3	3.5–23.1	<0.01

CI: confidence interval.

## Data Availability

Raw data were generated at the Tokyo Metropolitan Toshima Hospital. The data used in this study and the analysis code are available from the corresponding author upon request.

## Abbreviations

The following abbreviations are used in this manuscript:

WHO	World Health Organization
ANOVA	Analysis of variance
GMM	Gaussian mixture moder
BIC	Bayesian information criteria
